# Pro-environmental behavior regarding single-use plastics reduction in urban–rural communities of Thailand: Implication for public policy

**DOI:** 10.1038/s41598-024-55192-5

**Published:** 2024-02-27

**Authors:** Oluseye O. Oludoye, Nuta Supakata, Sarawut Srithongouthai, Vorapot Kanokkantapong, Stephan Van den Broucke, Lanrewaju Ogunyebi, Mark Lubell

**Affiliations:** 1 School of Health and Life Sciences, Teesside University, Middlesbrough, TS1 3BX UK; 2 Department of Environmental Science, Faculty of Science, Chulalongkorn University, Bangkok, 10330 Thailand; 3 Research Unit (RU) of Waste Utilization and Ecological Risk Assessment, Chulalongkorn University, Bangkok, 10330 Thailand; 4 Psychological Sciences Research Institute, Université Catholique de Louvain, Louvain-La-Neuve, Belgium; 5 Environmental Biology Unit, Department of Cell Biology and Genetics, Faculty of Science, University of Lagos, Akoka, Lagos, Nigeria; 6 Department of Environmental Science and Policy, University of California Davis, One Shields Drive, Davis, CA 995616 USA

**Keywords:** Environmental sciences, Environmental social sciences

## Abstract

The study investigates residents’ behavior towards reducing the use of single-use plastic (SUP), specifically in the context of food packaging. The widespread view holds that pro-environmental behavior (PB) results from a person’s moral and rational deliberations. In reducing single-use plastic (SUP) consumption and waste, the relative roles of rationality and morality models in validating PB among rural and urban residents are not yet clear. In this empirical study, we compared the relative efficacy of two models for explaining people’s SUP reduction behavior: the theory of planned behavior (TPB; rationality) and the value belief norm (VBN; morality). We investigated Thailand’s rural (Sichang Island) and metropolitan (Nonthaburi city) areas. As a result, we surveyed people living on Sichang Island (n = 255) and in Nonthaburi city (n = 310). We employed structural equation modeling (SEM) for data analysis in this study. Findings showed that while morality better justified all the study participants’ SUP reduction behavior, rationality underpinned behaviors of rural residents, while morality better explained the actions of city residents. We discussed future theoretical development and a policy roadmap based on these findings.

## Introduction

The food industry is the largest user of single-use plastics (SUP), accounting for up to 35% of global packaging production^1^. Almost 95% of food packaging is discarded after a single use^2^. SUP food packaging production, consumption, and management pose several environmental risks. A growing body of research focuses on the effects of plastics in marine, freshwater, and terrestrial environments^3–5^. SUP debris in the environment can degrade into smaller pieces known as microplastics (sizes less than 5 mm) and nanoplastics (1–100 nm)^6^, which can have various effects on wildlife and the health of coastal communities. Currently, plastic waste is considered a critical environmental issue in Thailand, with the country grappling with an ever-increasing volume of SUP waste^7^.

Thailand leads the Asian Pacific region in annual plastic consumption per capita, with 40 kg^8^. Every day, Bangkok alone produces 1800 metric tons of SUPs^9^. In Thailand, there has yet to be a significant impact on reducing plastic use. According to some estimates, about 12% of Thailand’s total municipal solid waste (MSW) is plastic^10^. SUPs waste accounts for a considerable portion of municipal solid waste (MSW). This proportion is rising due to lifestyle changes and is expected to increase further as MSW volumes have increased by about 10% per year^11^. According to a study^12^, urban residents have better plastic waste disposal habits than rural residents. The rural community’s custom of using plastic bags when shopping at accessible neighboring stalls exacerbates this. In many cities, particularly in developing countries, the waste management system designed by the government and private sector cannot overcome the number of existing landfills^13^. This prompted calls for a reduction in SUP food packaging waste to prevent increased amounts of plastic from land-based waste from entering the ocean. SUP can take up to 1000 years to decompose, which makes the situation worse, but consumers can dispose of it in a matter of minutes.

In recent years, the global environmental concern has escalated due to the extensive use of single-use plastic (SUP), particularly in the domain of food packaging. This study aims to contribute to our understanding of residents’ decision-making processes related to SUP reduction, with a specific focus on SUP used in food packaging. Changing people’s attitudes and behavior toward single-use plastic reduction is a difficult task that may take a long time to achieve. It is, however, one of the most fundamental methods of crisis mitigation^14^. Given that human behavior contributes to SUP littering, among other things, there is a need to gain insights into SUP perceptions, attitudes, and behaviors to develop better solutions that promote the reduction of these materials in the environment^15^. The 2020 pandemic crisis emphasized the importance of plastic items in food safety and human protection, but it also contributed significantly to the appearance of more plastic materials in the environment. Given the popularity of the SUP reduction campaign, plastic waste reduction behavior may have gained traction, but only among a subset of society^16^. The literature has abundant arguments for plastic waste management in rural and urban areas^17^. However, due to the rural areas’ role as a market for urban goods, there has been an increase in rural consumerism in recent times, necessitating a comparative examination of the situation with the urban areas^17^. Given those mentioned above, this study analyzed and compared the rational and moral antecedents of SUP reduction behavior between Sichang Island (rural) and Nonthaburi municipality (city) residents of Thailand. Other studies (e.g., tourism and climate change) have identified the rational and moral perspectives as antecedents of pro-environmental behaviors^18–21^, it is however unclear which of these two perspectives underpins people’s behavior to reduce SUP consumption in food packaging, especially in an urban–rural community. Therefore, the objective of this study is to examine and compare the effectiveness of the Theory of Planned Behavior (TPB) and Value–Belief–Norm (VBN) theory in explaining residents’ behavior towards reducing single-use plastic (SUP) in the realm of food packaging.

## Theoretical underpinnings

### Rationality and residents’ pro-environmental behavior

According to rationalist researchers, pro-environmental behavior (PB) is a reasoned choice in which a person weighs the personal benefits and drawbacks of their actions. Individuals are more likely to engage in PB if the benefits outweigh the costs of preserving and safeguarding the environment^20,22^. The theory of planned behavior (TPB) is the most prominent framework in this regard^21^. As Ajzen^23^ proposed, the TPB originated as an extension of the theory of reasoned action, in which volitional considerations primarily influence behavioral decisions. The TPB is a well-established cognitive-based theory that explains intentions and behavior in various contexts, including various pro-environmental activities^24^. The TPB predicted three determinants of intentions, the first of which is the attitude toward the behavior. The degree to which a person has a favorable or unfavorable evaluation or appraisal of the behavior is referred to as attitude. Subjective norms are the second determinant and are the extent to which a person feels social pressure to perform or refrain from performing a behavior. The third element is perceived behavioral control (PBC); it refers to the anticipated ease or difficulty while performing the behavior. Previous studies have applied the TPB and confirmed the usefulness of this model, especially in predicting SUPs reduction^18,25–28^.

### Modifying the TPB with situational factors

With regard to the use of the TPB in previous studies, the assumption is that rational decisions to use plastics are purely based on the volitional control of people, even though this may not always be the case^20^. This assumption overlooks the role of situational factors such as the perceived availability and cost of reusable alternatives among the residents. Therefore, the current study considers modeling the effects of such situational factors on the TPB to account for the extent to which these factors affect the rational decisions of the study participants. Previous studies indicated that situational factors significantly predict pro-environmental behavior. Among these factors, the cost and availability of facilities (reusable alternatives) provided causal reasons for these behaviors^20^. The high cost of reusable SUP alternatives, such as biodegradable plastics, hinders people’s behavior towards their use^29^. The low-cost hypothesis proposes that attitude affects ecological behavior most strongly when associated with low personal costs^30^. Past research has established that locals’ attitudes toward tourism development are influenced by the financial and social costs of doing so^31,32^.

**Figure 1:**
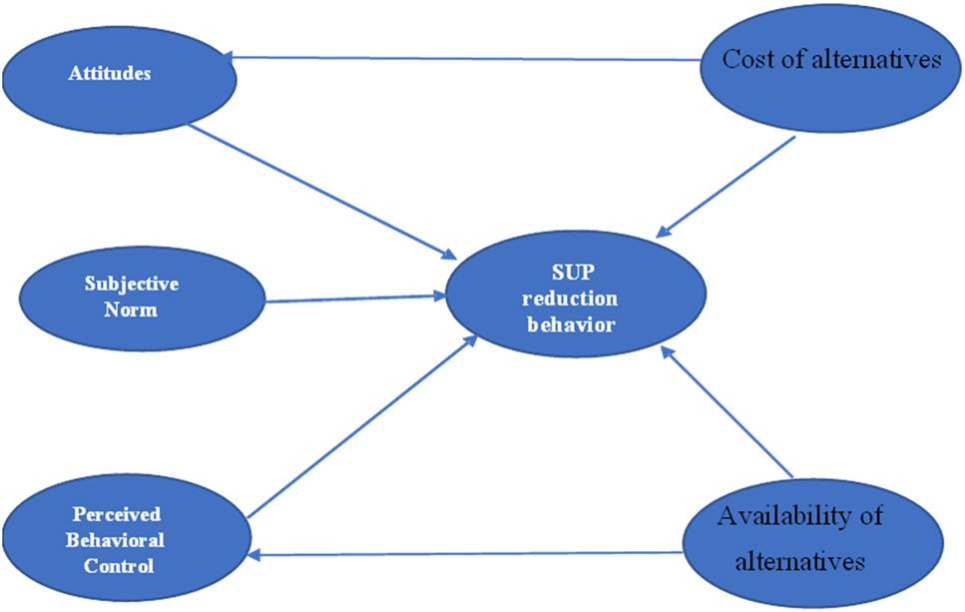
The modified Theory of Planned Behavior for this study. *Notes*: Subjective norms (SN), Perceived behavioral control (PBC), Attitudes (ATT), Availability of alternatives (AVA) and Cost of alternatives (COA).

Moreover, the provision of sufficient facilities was a vital determinant of waste management efficiency in municipals^33^. High satisfaction with local facilities would strengthen the residents’ PBC and behavior^33^, while a perceived lack of facilities would be a barrier^34^. In the context of SUP plastic reduction behavior, the availability of facilities (reusable alternatives) would facilitate and encourage individuals to voluntarily reduce their SUP consumption. Although different rational-choice models have been employed to describe PB, the TPB is primarily accepted as the most established and commonly used theory due to its clarity, efficiency, and competency. As a result, this paper employs a modified TPB model (Fig. 1) to investigate the role of rationality in understanding residents’ behavior regarding SUP reduction in Thailand, and it suggests the following hypothesis:

#### H1

The TPB is efficient in explaining SUP reduction behavior among Thai residents: i.e.,

#### H1a

Residents’ attitudes positively influence their SUP reduction behavior.

#### H1b

Residents’ subjective norms positively influence their SUP reduction behavior.

#### H1c

Residents’ PBC positively influence their SUP reduction behavior.

#### H1d

Cost of reusable alternatives negatively influence the residents’ SUP reduction attitudes.

#### H1e

Availability of reusable alternatives positively influence the residents’ SUP PBC.

### Morality and the residents’ pro-environmental behavior

**Figure 2:**
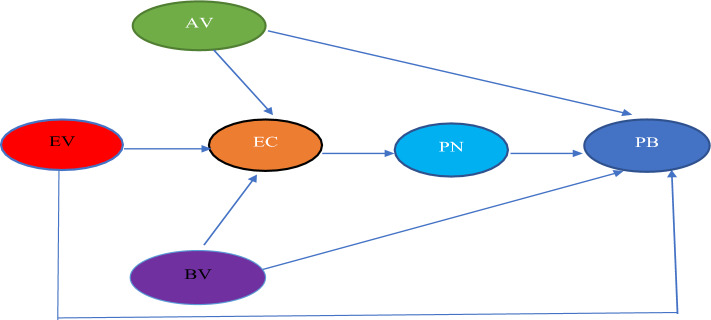
Value Belief Norm Theory^44^. *Note*: EV means egoistic values, AV means altruistic values, BV means biospheric values, EC means environmental concern, PN means personal norms and PB mean pro-environmental behavior.

According to "moralist" researchers, PB is a subset of prosocial behavior driven primarily by normative and moral considerations. One of the most common theoretical frameworks of moral obligation is the value belief norm (VBN) model^35^. The VBN theory, proposed by Stern and colleagues^36,37^, is an attempt to systematically combine the norm-activation model (NAM) theory from social psychology with the value-basis theory from environmental psychology and the environmental concern (or beliefs) from environmental sociology. The VBN model posits a causal chain in which social-psychological factors, such as values, beliefs, personal norms, and pro-environmental behavior, affect one another directly and may affect factors further along the chain (Fig. 2). According to the value-basis theory^38^, norms and behaviors are grounded in values concerned with the welfare of others (altruistic values), the interest of oneself (egoistic values), and the welfare of the ecosystem (biospheric values). Researchers have applied the VBN in PB, especially waste management^39^, coastal management^40^, energy conservation^41^, biodiversity^42^ and tourism^43^. Therefore, the following hypothesis is proposed:

#### H2

The VBN is efficient in explaining SUP reduction behavior among Thai residents:

#### H2a

Biospheric (BV) and altruistic values (AV) have a positive impact on SUP reduction behavior.

#### H2b

Egoistic values (EV) have a negative impact on SUP reduction behavior.

#### H2c

Biospheric (BV) and altruistic values (AV) have a positive impact on environmental concern (EC).

#### H2d

Egoistic values (EV) have a negative impact on environmental concern (EC).

#### H2e

Environmental concerns (EC) have a positive impact on personal norms (PN).

#### H2f

Personal norms (PN) have a positive impact on SUP reduction behavior.

### Comparing rationality and morality among residents’ pro-environmental behavior

According to Raghu and Rodrigues^35^, in some cases, a specific theory (e.g., TPB or VBN) is more successful than others in explaining PB, which demands additional research focus. These contextual determinants may include PB types^45^, PB consumption situations^46^, and participant demographics^47^. According to Steg and Vlek^48^, there are distinct conditions in which rational theoretical underpinnings are more successful in explaining antecedents to PB. In contrast, moral viewpoints may be more useful in other cases Steg and Vlek^48^. Though there is no agreement in the literature as to which of the two views is superior in describing people’s pro-environmental actions, some significant variations exist in their explanatory ability based on various contexts. For example, Aguilar‐Luzón et al.^49^ discovered that despite the TPB being a generic model for predicting and explaining behavior, the results show that it has a higher degree of fit and more capacity to predict recycling behavior than the VBN among Spanish homemakers.

### Discrepancies in pro-environmental behavior in rural–urban settings

In the rural-city context, rural residents, because of their place attachment to the village they live, are more likely to regard themselves as being in their home environment and hence bring their rational motives for engaging in pro-environmental activity with them^50–52^. Due to resource scarcity in rural residents, they tend to appraise the cost–benefit, behavioral outcomes or opinions of significant others before taking any pro-environmental actions. This ideology fits with the theory that rural dwellers are more likely to make sound choices than their urban counterparts, given their limited access to information and resources^53^. On the other hand, city residents have little or no relationship with the areas they live in, as most migrated from their villages to live and work in cities^54^. As a result, they do not see any direct benefits to themselves from acting pro-environmentally. Therefore, city residents participate in PB primarily for moral/ethical reasons, with the primary goal of conserving the environment rather than cost–benefit analysis, which underlies rural residents’ rationality^51,55^.

The current study focuses on the contextualized distinction between SUP reduction behavior between Nonthaburi municipality and Sichang Island residents of Thailand. Pro-environmental behavior’s decision-making process and underlying motives could differ between the residents mentioned above. These distinctions represent rationality and morality’s role in influencing their PB. This paper proposes that differences in decision-making processes and underlying reasons between the city and rural residents may alter the function of rationality and morality in eliciting PB. Therefore, the following hypotheses have been proposed:

H3: Compared with the VBN (morality), the TPB (rationality) is superior in explaining Sichang Island residents’ SUP reduction behavior.

H4: Compared with the TPB (rationality), the VBN (morality) is superior in explaining Nonthaburi municipality residents’ SUP reduction behavior.

The comparative analysis between Nonthaburi city and Sichang Island serves several key purposes such as enhancing generalizability of findings, informing targeted interventions and policies and refining exixsting theoretical frameworks. For example, analyzing variances in influencing factors helps advance theories such as the Theory of Planned Behavior (TPB) and Value–Belief–Norm (VBN), shedding light on contextual nuances that may influence residents’ behaviors differentially in urban and rural settings.

## Methods

### Study site and sampling

**Figure 3:**
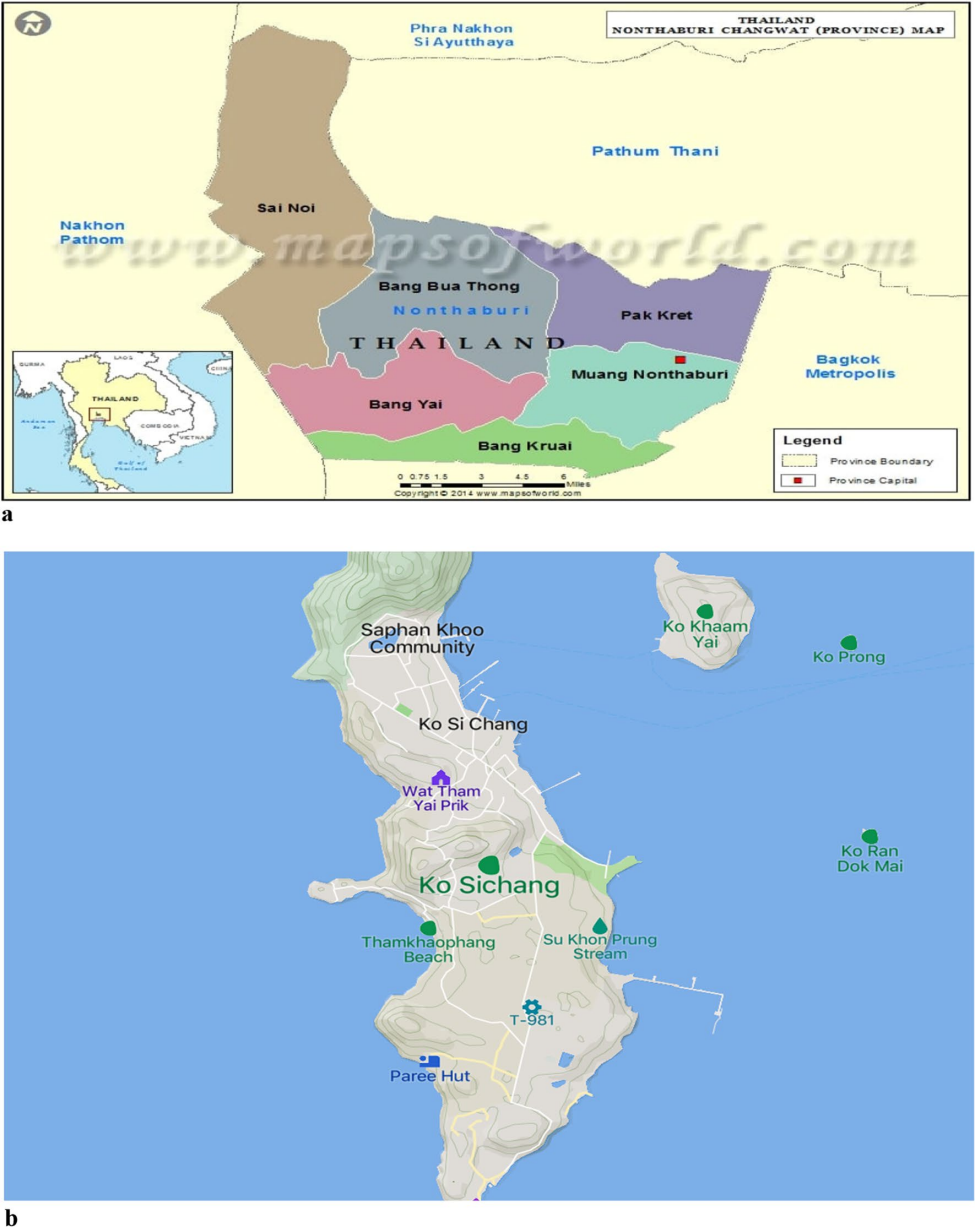
(**a**) Nonthaburi map^58^. (**b**) Sichang Island map^59^.

We chose Thailand’s Nonthaburi City Municipality (urban) and Si Chang Island (rural) as the study locations (Fig. 3a,b). The specific criteria for classification were based on population density, geographical size, and the effectiveness of waste management practices. These factors were chosen to capture the essential distinctions between urban and rural environments in the context of plastic waste management. There are 1,246,295 people living in Nonthaburi city, which has a total size of 622.38 square kilometers as of 2018^56^. The volume of waste dumped at the Nonthaburi Provincial Administration Organization (NPAO) disposal facility has grown from 2017 to 2019. 31.14% of the total waste disposed of in 2019 was categorized as plastic, rubber, and leather waste. The entire proportion had increased by 6.19% compared to the statistics examined in 2007, when the total amount of plastic, rubber, and leather waste was approximately 24.95%. The "Clean Province" strategy, which is a program to minimize and sort waste through public relations (3R policy) to reduce and stop the use of plastic bags, is the emphasis of plastic waste management in Nonthaburi. However, Koh Sichang (Sichang Island) is in an administrative boundary of Chonburi Province and consists of small islands located in the inner part of the eastern seaboard of the Gulf of Thailand. Sichang Island is characterized as rural based on factors such as its relatively small population size, limited urban infrastructure, and dependence on traditional livelihoods. Roughly 5000 people live in the village, totalling 17.3 square kilometres. Due to its small population, Sichang residents generate less solid waste, unlike Nonthaburi’s. However, microplastic contamination has been reported partly due to improper plastic waste management among the Island residents^57^. Additionally, Sichang Island faced challenges regarding the amount of garbage generated (approximately 10–15 tonnes per day) exceeding the incinerator’s capacity (able to burn six tonnes). As a result, stakeholder perspectives suggest sending garbage to the mainland and potential private-sector investments in incinerators as alternate waste management practices. We used purposive sampling to select participants who meet the study’s inclusion and exclusion criteria from each study location:

Inclusion criteria: Those who.

(i) were 18 years old or older during data collection.
(ii) can communicate in the Thai language.
(iii) were residents of Nonthaburi city and Si Chang Island.
(iv) use SUPs for food packaging.

Exclusion criteria: Those who.

(i) had a speech impairment
(ii) were foreigners or tourists,
(iii) use alternatives to SUPs
(iv) were not available for data collection

### Data collection

The municipality organized a seminar on plastic waste management to enlighten its residents on the danger of plastic to the environment. The municipality office invited the residents to participate in the seminar at the municipality office. About 350 residents attended the event, and 310, meeting our sampling criteria, agreed to participate in this study. Eight undergraduate/graduate students from Chulalongkorn University’s Department of Environmental Science, Faculty of Science, were recruited to assist with data collection. Before administering the questionnaires, the research assistants received one day of training. We obtained permission from the municipality office to collect data on the seminar day. We explained the study’s purpose to the participants, and their informed consent was obtained before data collection. We explained the questionnaire to the participants to aid their understanding of different concepts of the study via a big screen. We administered the questionnaire in the Nonthaburi municipality office’s conference hall.

For Sichang Island, we got the approval of the local authority (*thesaban* in Thai) before the study, like Nonthaburi city. We recruited three staff from the *thesaban* who acted as our guides in the village. This method helped us to establish trust and build rapport with the study participants. The questionnaire was distributed among the local participants using the above-mentioned inclusive/exclusive criteria with the assistance of research assistants. To increase the response rate, we adopted a face-to-face interview format for participants uncomfortable with self-administration. The research assistants interviewed each participant at their home or shops (where they sell) based on their choice. Before the interview, the assistants explained the questionnaire’s content to the participants to help them understand the various concepts in the questionnaire. Therefore, 255 of the 290 residents we visited agreed to participate in the study and were given the questionnaires to complete.

A nationally representative sample of 565 questionnaires was collected and completed correctly in both Nonthaburi and Sichang Island yielding a satisfactory response rate of 88%. The participants answered 48 questions comprising of sociodemographic factors, the TPB and VBN. The interview time for each participant ranged from 30 to 45 min. The questionnaire was administered in Thai in a self-report format. However, some participants preferred the questionnaire to be administered verbally in a face-to-face interview. The questionnaires were checked for completeness after the interview to confirm any missing information from the participants. In the total population sample, female respondents outnumbered male respondents by a margin of 56.8–42.4%, and the average age of the residents was 41.23 years. Most respondents (55.5%) were single and educated (55% had at least a primary school certificate). The trend is also the same in Nonthaburi city municipality and Sichang Island regarding the participants’ average age, gender, and marital and education status. In Nonthaburi city municipality, there are more female participants (56.4%) than male (42.8%) participants, the average resident age was 41.88 years, and most of them were single (59.2%) and educated (68.9% with a least a primary school certificate). In Sichang Island, female respondents (57.2%) were also more than the male respondents (41.9%), their average age was 40.51 years, and most of them were single (51.5%) and educated (51.2% with at least a primary school certificate). Therefore, this study’s sociodemographic findings show our sample’s representativeness as it is consistent with the general Thai population data of the National Statistical Office (NSO) of Thailand^60^.

### Measures

We divided the questionnaire into four major sections (Table 1). The first section included an informed consent form that participants must sign before proceeding. The second section had demographic questions for the participants. The third section focused on the participants’ SUP reduction behaviors and questions designed to assess the TPB and VBN constructs. The agreement or disagreement on the subject was measured using a five-point Likert scale (1 = strongly disagree to 5 = strongly agree).

**Table 1:** Description of variables considered in the study.

Variable*	Meaning
The TPB	
Attitudes	It refers to either positive or negative behavioral assessment and outcome
Subjective norms (SN)	It is the person's perception that others who are important to them want or do not want them to carry out that particular behavior
PBC	It refers to a person's perception of difficulty or ease in carrying out a behavior
Availability of alternatives (AVA)	It refers to various packaging alternatives that can replace single-use plastic food packaging. This item was designed to gauge perceptions and attitudes toward the accessibility of alternatives among those who primarily use SUP for food packaging
Cost of alternatives (COA)	It means the price of reusable packaging and the affordability of alternatives
The VBN	
Biospheric values (BV)	Those values that focus on the state of the environment align with the belief that protecting the environment is an essential goal in life
Egoistic values (EV)	A value orientation that puts self-interest at the center of individual decision-making rather than behavior that favors the environment. i.e. values that focus on maximizing personal benefits over behaviors that benefit the environment
Altruistic values (AV)	It refers to collective values about people and creatures that encourage people to engage in environmentally sound behaviors
Environmental concerns (EC)	It is an individual’s perception of the environment's vulnerability due to human intervention, i.e., the violation of humanity's environment and the earth's limited resources
Personal norms (PN)	It refers to self-expectation (internal values) to demonstrate certain behaviors
SUP reduction behaviors	It refers to actions or practices to avoid or reduce the use of SUPs as food packaging materials

The attitude toward SUP reduction behaviors was adapted from the work of Adam^20^. The Study of Goh et al.^61^ was used to develop subjective norm measures. The measures for behavioral control were adapted from the work of Soomro et al.^62^ and Rastegari Kopaei et al.^63^. The VBN theory assessed participants’ values by modifying the eight subscales from Schwartz’s latest Portrait Values Questionnaire (PVQ-RR)^64^. The revised New Ecological Paradigm Scale was used to assess environmental concern (EC), i.e. beliefs^65^. Personal norms were used to evaluate norms, and the questionnaire items were adapted from Steg et al.^66^. The situational factors, availability and cost of alternatives were modified from the study of Leung and Rosenthal^67^. The VBN items were rated as opposed to my guiding principles (1), not important (2), important (3), very important (4), and of supreme importance (5). For content validity of the questionnaire, a panel of environmental science and psychology experts (n = 5) reviews a draft version of the questionnaire for content validity. Before it was used for data collection, it was modified based on their feedback. A pilot study was conducted with a small sample of participants (n = 30) to assess the internal consistency of the questionnaire using Cronbach’s alpha coefficients (α). The results indicated that the questionnaire subscales were sufficiently reliable (α ≥ 0.7).

### Data analysis

All data were checked for missing values before analysis. Data from questionnaires were coded, cleaned, and checked for outliers using Excel software. To avoid poor model fit, outliers were removed. The data were imported into the Statistical Package for Social Sciences (SPSS) software (version 28) for further analysis. The data were analyzed using exploratory factor analysis (EFA), confirmatory factor analysis (CFA), and structural equation modelling (SEM) with AMOS version 22. In this regard, we evaluated the model’s goodness-of-fit, convergent validity, and reliability. Numerous indices have been proposed as measures of model goodness of fit and were used in this work. They are the relative or normed Chi-square (χ^2^/df), the comparative-fit index (CFI), the incremental fit index (IFI), the goodness-of-fit index (GFI), and the root mean squared error approximation (RMSEA). The structural model was also used to find a best-fitting model and causal linkages between variables (Path Analysis).

### Ethics approval

The study was approved by Chulalongkorn University Research Ethics Committee (COA No. 032/66). Moreover, we obtained informed consent from all the participants before the interview concerning the study objectives, procedures and benefits. In addition, all methods in the manuscript were performed in accordance with the relevant guidelines and regulations of the Declaration of Helsinki.

## Results

### Validity and reliability assessment

An EFA was first performed to evaluate the factorial structure of the measurement items. Tables 2 and 3 indicate that all the items had significant loadings and were loaded unto only one rotational factor^68^. Every unobserved variable, however, has a robust Cronbach alpha coefficient larger than 0.70^69^. The CFA was carried out to validate the measurement validity of the items. All the elements from the CFA model had good loadings greater than 0.50^68^. The study data fit the model well, according to the model fit indices (GFI = 0.933; CFI = 0.937; IFI = 0.971; RMSEA = 0.044) of the measurement model^70^. Furthermore, all unobserved variables have high composite reliability (CR) coefficients greater than 0.70. These results suggest that the observed variables accurately captured the respective unobserved variables^71^. The average variance extracted (AVE) and the standardised estimates’ size and significance were evaluated to determine the convergent validity of the measuring items. Tables 2 and 3 show that all standardized values are higher than 0.50 and statistically significant (*p* ≤ 0.01). The validity of the unobserved variables is supported by the fact that each unobserved variable’s AVE is larger than 0.50^71^.

**Table 2:** The results of the EFA and CFA for the TPB model (n = 565).

Factors	EFA	CFA		
	Loadings	Standardized coefficients	CR	AVE
Attitudes (Cronbach α = 0.891)			0.879	0.771
I believe that reducing SUPs is necessary to protect the environment	0.887	0.894		
SUPs, in my opinion, are harmful to the coastal environment	0.918	0.883		
SUPs are, in my opinion, hazardous to human health	0.881	0.901		
I believe that using reusable alternatives to reduce SUPs is worthwhile	0.832	0.911		
Subjective Norms (Cronbach α = 0.913)			0.809	0.718
Most important people in my life will support my decision to use SUPs	0.818	0.886		
Most people I know would approve of me paying to use reusable alternatives to SUPs	0.887	0.889		
I use SUP to gain the approval of my friends	0.831	0.843		
Perceived Behavioral Control (Cronbach α = 0.801)			0.927	0.788
It is easy for me to decline free SUPs and pay for reusable alternatives	0.788	0.889		
It is convenient for me to carry SUPs instead of taking reusable alternatives	0.773	0.868		
Whether I reduce SUPs completely depends on me	0.819	0.899		
Reducing SUPs is a difficult task	0.855	0.880		
Availability of alternatives (Cronbach α = 0.910)			0.930	0.707
There are enough reusable alternatives to SUPs	0.833	0.898		
The opportunities for SUPs reduction are well outlined by the government	0.911	0.970		
There are eco-friendly alternatives to SUPs in food packaging	0.889	0.877		
Cost of alternatives (Cronbach α = 0.961)			0.912	0.730
Reusable alternatives are more expensive than SUPs	0.843	0.883		
Biodegradable alternatives are more affordable than SUPs	0.776	0.885		
The cost of reducing the unnecessary use of SUPs is unaffordable	0.898	0.833

**Table 3:** The results of the EFA and CFA for the VBN and SUP reduction behavior (n = 565).

Factors	EFA	CFA		
	Loadings	Standardized coefficients	CR	AVE
Biospheric Values (Cronbach α = 0.811)			0.890	0.670
Preventing pollution	0.810	0.933		
Respecting the earth; living in harmony with other species	0.893	0.886		
Unity with nature; fitting into nature	0.919	0.858		
Protecting the environment; preserving nature	0.850	0.800		
Egoistic Values (Cronbach α = 0.918)			0.887	0.772
Leading or commanding people (Authority)	0.899	0.904		
Having material possessions or money (Wealth)	0.770	0.898		
Having control or dominance over others (Social power)	0.897	0.904		
Having an impact on people and events (Influential)	0.890	0.840		
Altruistic Values (Cronbach α = 0.977)			0.884	0.691
A world at peace; free of war and conflict	0.879	0.887		
Equality; equal opportunity for all	0.899	0.894		
Helpful; working for the welfare of others	0.789	0.881		
Social justice; correcting injustice, caring for the weak	0.712	0.900		
Environmental Concern (Cronbach α = 0.939)			0.933	0.775
The so-called "ecological crisis" facing humankind has been greatly exaggerated	0.913	0.803		
The earth is like a spaceship with limited room and resources	0.888	0.884		
The balance of nature is strong enough to cope with the impacts of modern industrial nations	0.896	0.829		
Mankind is severely abusing the environment	0.887	0.838		
Personal Norms (Cronbach α = 0.883)			0.899	0.700
I am morally obligated to protect the environment by using reusable alternatives to SUPs	0.779	0.847		
Taking action toward environmental protection is in line with my moral principles	0.842	0.859		
I would feel guilty if I did not do anything to protect the environment by avoiding SUPs	0.854	0.828		
Our environmental problems cannot be ignored	0.868	0.837		
Actual Behaviors (Cronbach α = 0.985)			0.938	0.787
I refuse plastic shopping bags by using reusable alternatives	0.891	0.849		
I avoid buying bottled water by using personal reusable bottles	0.900	0.919		
I avoid prepackaged fruits and vegetables by choosing loose produce	0.914	0.903		
I refuse straws with takeaway coffee cups or drinks	0.710	0.817		
I choose eco-friendly alternative materials, even if they are expensive	0.880	0.849		

### Rational and moral model testing with the total residents’ sample

The entire sample (n = 565) was evaluated to determine how well the modified TPB and VBN performed overall in predicting the participants’ SUP reduction behavior using the SEM. The performance of the two models was assessed using two incremental fit measurements (CFI and IFI) as well as three absolute fit measurements (χ^2^/df, GFI, and RMSEA). A model’s fit is considered good when the χ^2^/df is below 3, and the RMSEA is below 0.08^68^. The GFI, CFI, and IFI have values between 0 and 1, and values greater than 0.9 signify a good model fit^68^. According to SEM’s goodness-of-fit test, both of the two models (TPB and VBN) adequately describe the data (TPB: χ^2^/df = 3.30, RMSEA: 0.07, GFI: 0.91, CFI: 0.95, IFI: 0.90; VBN: χ^2^/df = 2.92, RMSEA: 0.05, GFI: 0.97, CFI: 0.94, IFI: 0.92) (Table 4).

**Table 4:** Goodness-of-fit indices of the sample.

Fit indices	Sichang Island (n = 255)		Nonthaburi city (n = 310)		Total sample (n = 565)	
	TPB	VBN	TPB	VBN	TPB	VBN
χ^2^/df	2.91	3.98	3.17	2.88	3.30	2.92
GFI	0.96	0.92	0.90	0.96	0.91	0.97
CFI	0.95	0.93	0.93	0.95	0.95	0.94
IFI	0.93	0.91	0.91	0.94	0.90	0.92
RMSEA	0.06	0.08	0.09	0.07	0.07	0.05

**Figure 4:**
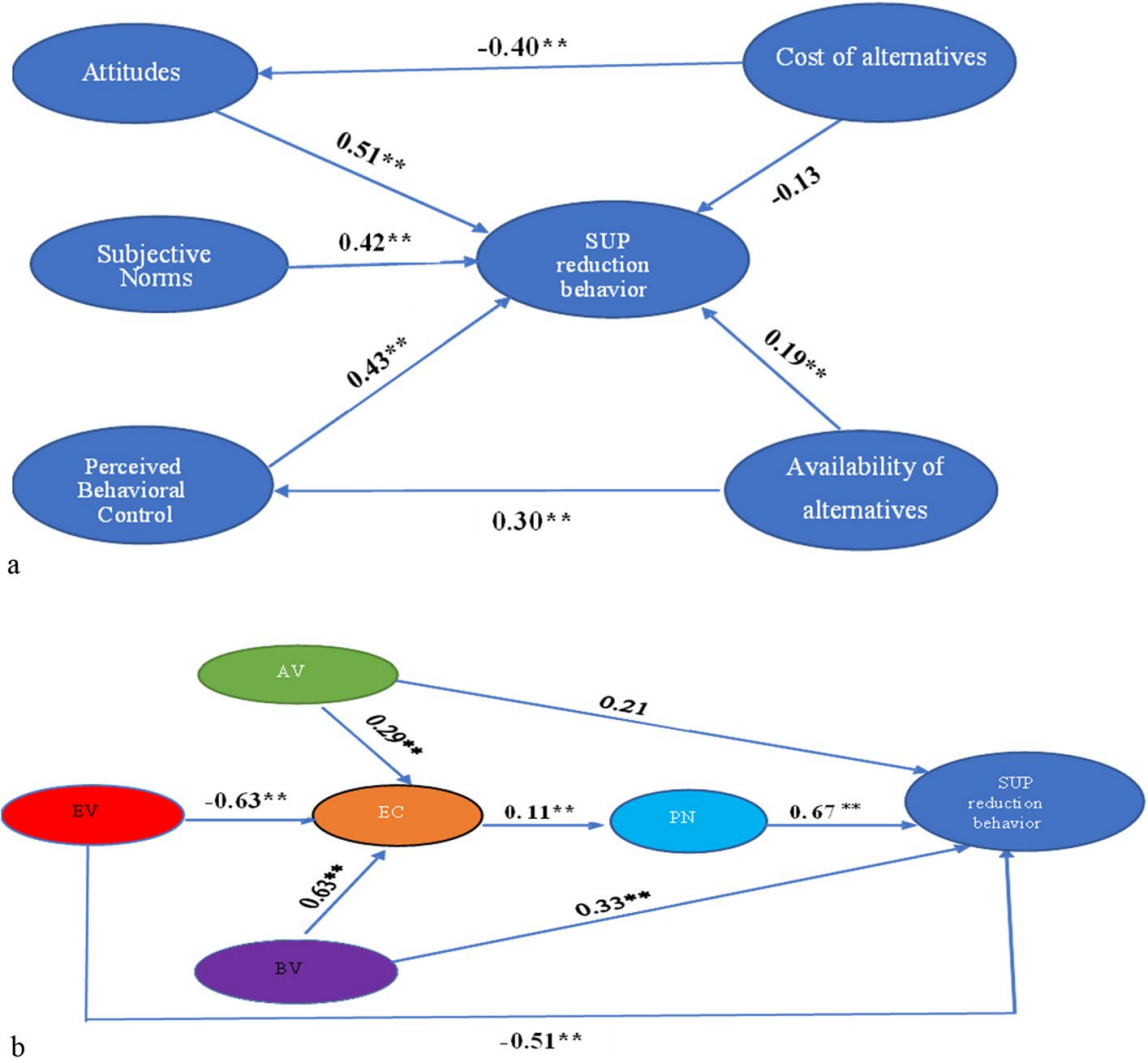
(**a**) The modified TPM model of all the participants using path analysis. (**b**) The VBN model of all the participants using path analysis.

Except for the relationship between AV → SUP reduction (β = 0.21, *p* > 0.01) and cost of alternatives → SUP reduction (β =  − 0.13, *p* > 0.01) in the VBN model, all of the standardized path coefficients among the variables in TPB (Fig. 4a) and VBN (Fig. 4b) were positively significant (*p* < 0.01). Thus, both the TPB and the VBN models successfully explain residents’ PB to reduce SUPs. However, when comparing the two model fit indices, the VBN model outperforms the TPB model. Additionally, we assessed the variance explained by the antecedents of the TPB and VBN regarding SUPs reduction behaviors. The TPB’s antecedents explained 35.7%, while the VBN’s antecedents explained 51.2% of the total variance in the residents’ SUP reduction behavior. Therefore, the SUPs reduction behavior is better explained by the VBN than the TPB when considering both the model fit indices and the variation explained.

### Models’ comparison between Nonthaburi city and Sichang Island residents

**Figure 5:**
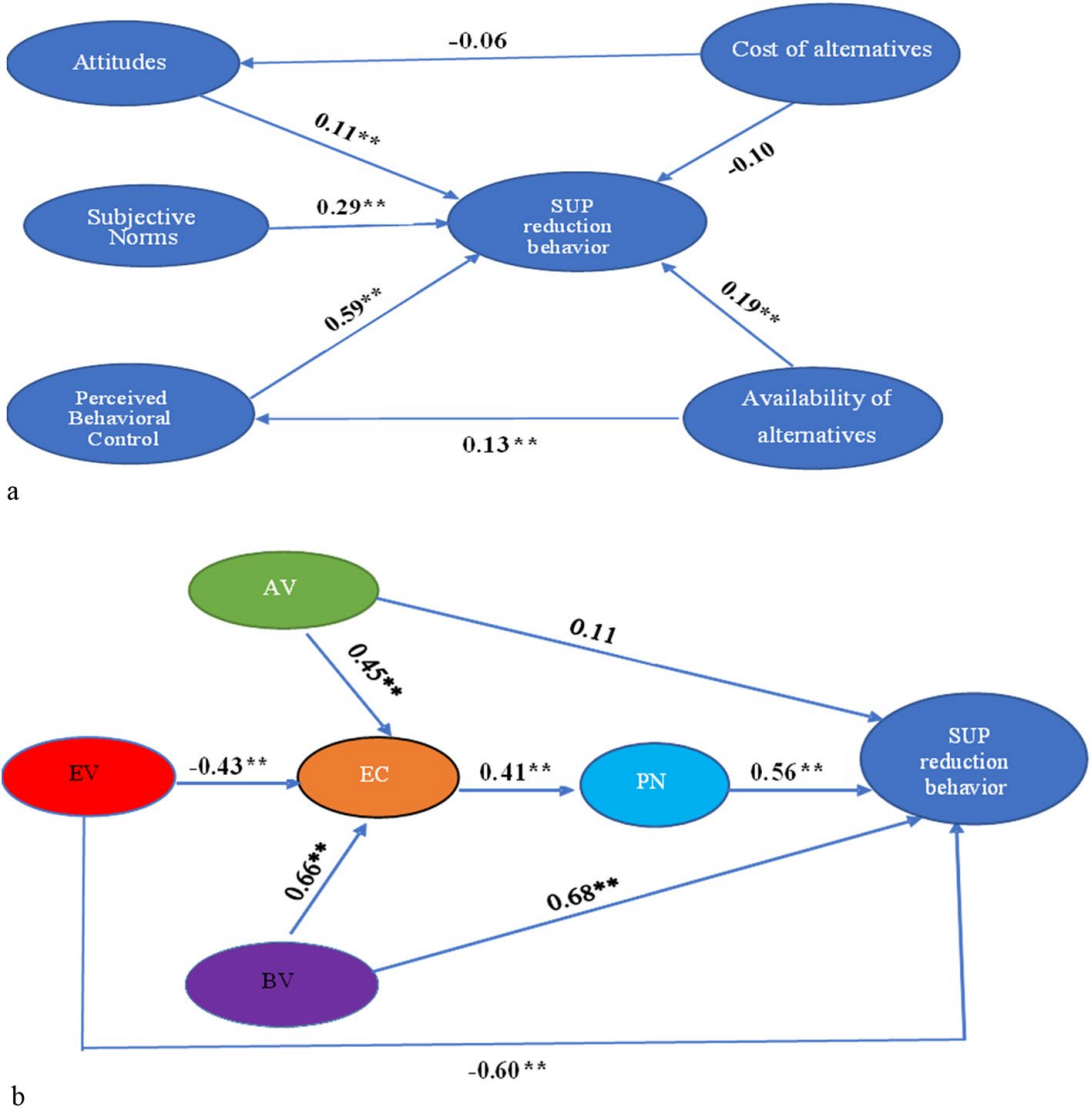
(**a**) A path analysis of Nonthaburi municipality residents’ antecedents of the TPB. (**b**) A path analysis of Nonthaburi municipality residents’ antecedents of the VBN.

The VBN had higher model fit indices (χ^2^/df = 2.88, RMSEA = 0.07, IFI = 0.94, CFI = 0.95, GFI = 0.96) than the TPB (χ^2^/df = 3.17, RMSEA = 0.09, IFI = 0.91, CFI = 0.93, GFI = 0.90) (Table 4) for the Nonthaburi city municipality residents, according to the data. Furthermore, while the TBP explained 30.3% of the variance in the SUPs reduction behavior, the VBN accounted for 48.9% of the total variance. The findings indicate that morality has a greater explanatory ability than TPB for predicting Nonthaburi municipality residents’ PB regarding SUPs reduction. In the TPB, all the paths were significant except the cost of alternatives to attitudes and SUP reduction behavior (Fig. 5a). While all of the causal paths were also significant, only the paths from AV → SUP reduction behavior were statistically insignificant (β = 0.11, *p* > 0.01) in the VBN (Fig. 5b).

**Figure 6:**
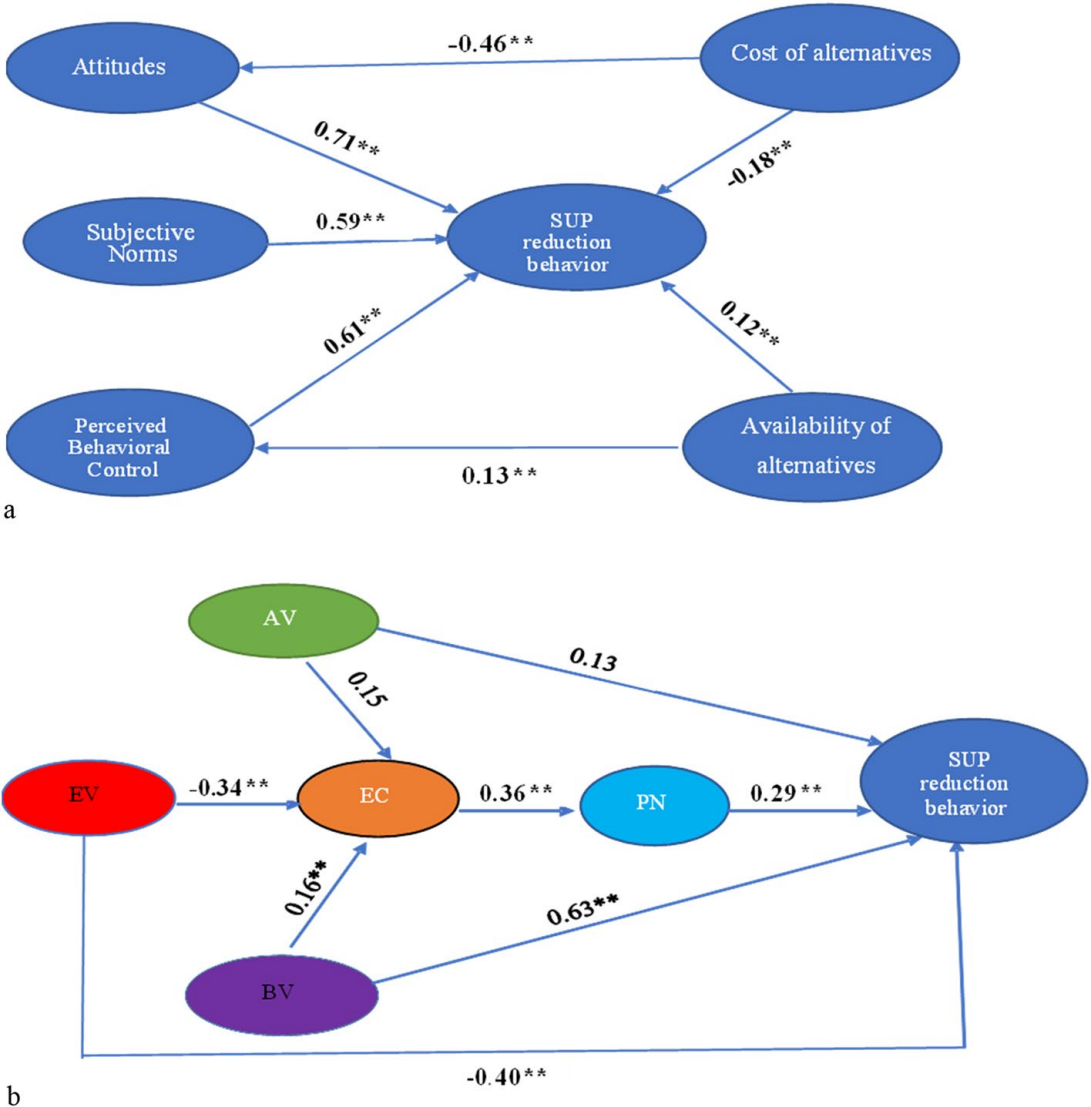
(**a**) A path analysis of Sichang Island residents’ antecedents of the TPB. (**b**) A path analysis of Sichang Island residents’ antecedents of the VBN.

The modified TBP model provides superior model fit indices (χ^2^/df = 2.91, RMSEA = 0.06, IFI = 0.93, CFI = 0.95, GFI = 0.96) for Sichang Island residents than the VBN model (χ^2^/df = 3.98, RMSEA = 0.09, IFI = 0.91, CFI = 0.93, GFI = 0.92) (Table 4). Furthermore, the modified TBP has a higher variance explained (57.1%) than the VBN (39.7%). Compared to the VBN, the modified TPB and, by extension, rationality has a stronger explanatory power among Sichang Island residents. All potential paths in the modified TPB model were statistically significant (Fig. 6a). In the VBN model, however, all pathways except two were positively significant (Fig. 6b); in particular, the paths from AV → EC (β = 0.15; *p* > 0.01) and AV → SUP reduction behavior were insignificant (β = 0.13, *p* > 0.01).

## Discussion

Scholars from various disciplines have regularly evaluated the effectiveness of rational or moral models in explaining pro-environmental behaviors^20–22,72^. Nevertheless, the literature has frequently questioned whether each perspective is sufficient^35,48^. This study adopted the modified TPB and VBN as representatives of the two perspectives, and it examined their relative strengths in explaining SUP reduction behavior among city and rural residents of Thailand. According to our findings, while the modified TBP and VBN are essential in explaining why residents choose to reduce SUPs in food packaging, the VBN, based on moral theorization, does so more effectively. This outcome contributes significantly to residents’ plastic-use reduction goals’ rational and ethical theoretical foundations. Therefore, the reduction behavior of all the residents regarding SUPs is more of a moral choice than a rational one. Reducing SUPs can be seen as having prosocial behaviors with a normative goal to preserve the environment for the benefit of others^73^. The finding further supports the idea that, among residents, morality and, to some extent, a sense of obligation weighs more heavily than the idea of rational considerations that support rational theorization in terms of actions geared toward protecting and preserving the environment^74^.

Compared to moral motivations, rational explanations were a greater predictor of Sichang Island residents reducing SUPs than were moral motivations for Nonthaburi municipality residents in this study. Even though both the TBP and VBN models are pertinent to explaining residents’ behaviors regarding SUPs reduction, how effectively they can do so depends on whether the residents are rural or city dwellers. Given that Sichang Island residents are rural dwellers, one can argue that they may have a strong sense of place attachment to their coastal environment. As a result, their decision to reduce SUPs may be motivated by their interests, such as improving their local environment and social status^45^. Therefore, the residents are more likely to weigh the merits and demerits of SUP reduction in their everyday lives and, consequently, to be motivated by rationality. To support this argument, the antecedents of the TPB in Sichang Island, especially attitudes, subjective norms and situational factors (cost and availability of reusables), were more robust than that of Nonthaburi city.

Regarding attitude, the literature demonstrates its favorable effect on environmentally conscious behavior^20^. Residents that perceive SUP as an environmental threat have a positive disposition toward its decrease or use of alternatives. In terms of subjective norm, its direct favorable influence on SUP reduction behavior implies that important others, such as family and friends, perceive SUP consumption reduction as a sustainable solution to the environmental crisis brought by SUPs. Also, residents who perceive reusable alternatives as costly and unavailable are less likely to reduce SUP, irrespective of their positive attitudes and perceived behavioral control. The less cost pressure there is, the easier it is for people to change their behavior to match their attitudes^30,75,76^.

One can argue that Nonthaburi municipality residents’ values, beliefs, and personal norms underlie their behavior regarding SUP reduction. As a result, their actions are based on reducing their plastic use to protect the marine ecosystem and environment^48,66^. Their actions to reduce SUPs may be interpreted as an example of prosocial behavior and may therefore be motivated by moral considerations related to the VBN rather than rational considerations of the TBP^48^. Thus, the normative goals may lead Nonthaburi residents to believe that saving the environment by reducing plastic consumption in food packaging is just the right thing to do. Compared to the rural residents (Sichang Island), the majority of city residents from this survey are more aware of the government policies aimed at reducing the consumption of SUPs^77^. Hence, Nonthaburi residents may have more pro-environmental norms, values, and orientation, which motivates them to act in a pro-environmental manner. To corroborate the above assertions, the antecedents of VBN in Nonthaburi city, especially biospheric and egoistic values, were more potent in predicting environmental concerns and SUP reduction behavior than Sichang Island. These findings, consistent with those of earlier research^78^, suggest that residents whose actions are motivated by self-interest are less likely to engage in SUP reduction. Conversely, pro-environmental behavior is more common among residents who place environmental well-being at the center of their lives^79^. This result suggests that people who care about the biosphere’s health have more positive views of human-environmental relations and greater concern for the natural environment^44^.

## Study implications

### Theoretical implications

To the best of the authors’ knowledge, this is one of the first studies to evaluate the effectiveness of rationality- and morality-based theories in simultaneously explaining residents’ behavior in a plastic-use reduction in cities and rural locations. This study’s findings contribute to the literature in various respects. First, it demonstrated that the TPB and VBN explain individuals’ pro-environmental decisions in either cities or villages. The study’s findings of the VBN’s greater explanatory power among the residents (total sample) suggest that the VBN should be employed in conceptualizing and explaining people’s pro-environmental behaviors because it has better predictive validity than the TPB. One would be able to comprehend residents’ behavior better to engage in other pro-environmental activities in this manner than using rational models only, particularly the TPB. The study’s findings revealed that the applicability of the modified TBP and VBN differs between rural and city residents. These findings suggest that future research on residents’ pro-environmental behaviors should avoid using a single model (one-size-fits-all) for all the residents, irrespective of their locations. The outcomes of this study support the notion that rural (Sichang Island) residents are best examined from a rational viewpoint. Still, city residents (Nonthaburi) are better understood from a moral standpoint. Also, the study findings imply that the TPB model should be extended to include paths from availability and the cost of reusable alternatives when conceptualizing people’s pro-environmental behaviors. The addition of these new paths was significant, and their future inclusion may improve the TPB predictive ability, especially as it explains how the cost and availability of reusable alternatives may influence SUP reduction.

### Practical implications

Microplastic pollution of environmental matrices is widespread in Thailand, caused by improper plastic waste disposal. As a policy framework and direction to solve this issue, "Thailand’s Roadmap on Plastic Waste Management 2018–2030" was developed by the government. One of the roadmap’s targets is to reduce and replace SUP with environmentally friendly and reusable alternatives. Therefore, this study’s findings have several practical implications for the roadmap, especially for the food service sector’s stakeholders (consumers, grocery stores and government sectors).

First, government sectors should segment the population/consumers based on their urban–rural differences before any policy interventions. Then they may use commercials and other communication methods to target each group with pro-environmental messaging specifically. For example, pro-environmental messages that appeal to their sense of reason may focus on rural residents. In contrast, messages that appeal to their moral values (mainly biospheric values) could be targeted at urban dwellers. In the cities, on-site education initiatives should prioritize environmental sustainability. It is essential to educate locals on the current environmental crisis and instill in them a sense of accountability to act sustainably. In rural areas, regulatory tactics targeting residents’ attitudes, social norms and perceived behavioral control could be more effective and should be implemented to encourage participation in PB. This regulation may include incentives (such as discounts and subsidies) for consumers to reduce the cost of environmentally friendly alternatives. Also, the authority should make reusable alternatives available in specific areas by establishing return points with incentives and without charging additional service fees. In educating the residents, household heads or influential people should be used as peer educators to other members of the communities.

Being the significant contributors of SUPs, grocery businesses or stores should actively minimize the number of SUP products they provide in favor of more affordable reusable alternatives. In this regard, grocery stores can reduce some SUP products (disposable straws, cups, and plastic beverage bottles) and replace them with reusable alternatives. Under product substitution strategies, the introduction of reusable alternatives such as straws, cups, and beverage containers is proposed. These alternatives are tailored to align with consumer preferences. This approach ensures they are accessible to residents and ready for use when the targeted messages prompt them to reduce SUPs consumption. Additionally, city officials and administrators of rural areas can provide training and assistance to locals to participate in creating and supplying reusable alternatives. These collaborative initiatives are encouraged, particularly through community awareness campaigns. By collaborating with local communities, these campaigns aim to raise awareness among residents about the environmental impact of SUPs and promote the benefits of adopting alternative, eco-friendly practices. Given the aforementioned, grocery businesses can purposefully raise the price of some SUP products so that residents can pay more to buy them. This tactic would increase the price of SUPs and deter some residents from purchasing them, especially rural residents whose pro-environmental objectives are based on sound reasoning to avoid doing so. In terms of pricing tactics, on the other hand, the study suggests the implementation of loyalty programs by grocery stores. This involves offering benefits to customers who consistently opt for reusable products, incentivizing sustainable choices.

## Conclusions

This study assessed and compared the applicability of TPB and VBN in SUPs reduction behavior among rural–urban residents of Thailand for the first time. The study concluded that moral antecedents of the VBN rather than rational antecedents of the TPB more effectively account for all the participants to reduce SUPs in food packaging. The study’s findings show that the VBN had more variance explained for the entire sample and superior model fit indices. We concluded that moral rather than rational antecedents provide a more compelling explanation for Nonthaburi city residents’ behaviors regarding SUP reduction. Sichang Island residents’ propensity for reducing SUPs in food packaging is determined by how they perceive the advantages or disadvantages it provides, approval from influential people around them and the cost and availability of alternatives. The study also concludes that moral reasoning provides a better explanation for why city residents adopt SUP reduction activities.

## Data Availability

The authors confirm that the datasets used in the current study are available from the corresponding author upon reasonable request.
